# The Predictive Value of Inflammation-Related Peripheral Blood Measurements in Cancer Staging and Prognosis

**DOI:** 10.3389/fonc.2018.00078

**Published:** 2018-03-21

**Authors:** Joanna L. Sylman, Annachiara Mitrugno, Michelle Atallah, Garth W. Tormoen, Joseph J. Shatzel, Samuel Tassi Yunga, Todd H. Wagner, John T. Leppert, Parag Mallick, Owen J. T. McCarty

**Affiliations:** ^1^VA Palo Alto Health Care System, Palo Alto, CA, United States; ^2^Biomedical Engineering, School of Medicine, Oregon Health & Science University, Portland, OR, United States; ^3^Canary Center at Stanford, Department of Radiology, Stanford University School of Medicine, Stanford, CA, United States; ^4^Department of Radiation Medicine, Oregon Health & Science University, Portland, OR, United States; ^5^Division of Hematology and Medical Oncology, Oregon Health & Science University, Portland, OR, United States; ^6^Cancer Early Detection & Advanced Research Center, Oregon Health & Science University, Portland, OR, United States; ^7^Knight Cancer Institute, Oregon Health & Science University, Portland, OR, United States; ^8^Department of Surgery, Stanford University School of Medicine, Stanford, CA, United States; ^9^Department of Urology, Stanford University School of Medicine, Stanford, CA, United States

**Keywords:** neutrophil-to-lymphocyte, platelet-to-lymphocyte, C-reactive protein, prognosis, cancer, longitudinal, biomarkers

## Abstract

In this review, we discuss the interaction between cancer and markers of inflammation (such as levels of inflammatory cells and proteins) in the circulation, and the potential benefits of routinely monitoring these markers in peripheral blood measurement assays. Next, we discuss the prognostic value and limitations of using inflammatory markers such as neutrophil-to-lymphocyte and platelet-to-lymphocyte ratios and C-reactive protein measurements. Furthermore, the review discusses the benefits of combining multiple types of measurements and longitudinal tracking to improve staging and prognosis prediction of patients with cancer, and the ability of novel *in silico* frameworks to leverage this high-dimensional data.

## Introduction

Cancer-associated inflammation is known to occur in the tumor microenvironment and in the systemic circulation and is correlated with disease progression and prognosis in many cancers (1). Immune mediators are involved in many phases of cancer progression including carcinogenesis, tumor growth, tumor invasion, and metastasis (2–5), and in turn, cancer cells can recruit and activate immune cells *via* direct contact (6) and/or through production of chemokines (7, 8) and prostaglandins (9). Numbers of circulating blood cells including neutrophils, lymphocytes, platelets, and levels of circulating proteins including C-reactive protein (CRP) and interleukins (ILs) associated with inflammatory responses, are key factors in recognition of pathways for tumorigenesis and growth (10) and could provide valuable information for improved cancer patient risk stratification and more targeted patient care.

Methods to improve early detection of cancer and gauge cancer patient prognosis are essential. Early detection can help identify cancers when they are still in a curative stage. Prognostic factors help to inform patients and physicians in end-of-life decision-making and can be utilized as inclusion/exclusion criteria for aggressive therapies or clinical trials. Overall, diagnostics and prognostics are critical to cancer therapy, and despite advances in technology and understanding of cancer cell biology, there are still many unmet needs. Currently, methods to predict tumor risk by investigating histopathological and clinical evidence such as tumor size, histological grade, histological subtype, or age are limited, and often fail to accurately stratify low- and high-risk patients (11–13). Tumor characteristics alone do not sufficiently determine patient outcomes in many malignancies likely due to heterogeneity in the patients, their innate immune response, and the genetic drivers of the disease. One way to integrate measurements of systemic immunity would be to include inflammation-associated cell enumeration, which can be easily obtained with a complete blood cell (CBC) count. The CBC count, the most frequently ordered laboratory test, consists of an automated hemogram and a five-cell automated differential count (14). The measurements in a CBC count include the white blood cell count consisting of neutrophils, lymphocytes, monocytes, eosinophils, and basophils, red blood cell count, hematocrit, hemoglobin, red blood cell indices (mean corpuscular volume, mean corpuscular hemoglobin, and mean corpuscular hemoglobin concentration), platelet count, and mean platelet volume. Select peripheral blood measurements also record information on important inflammatory markers such as CRP. Together, these components may have potential for prognosis in cancer patients. Compared to more invasive traditional diagnostic and staging tests, such as tumor size, histological grade, vascular invasion, lymph node metastases that would require surgery or expensive imaging techniques, a CBC, *via* a blood draw, is low cost and can be performed on a regular basis, with minimal risk to the patient.

An increasing body of evidence provides rationale for the utility of peripheral blood tests to predict cancer patient prognosis and treatment effectiveness. For example, a recent organism-wide study demonstrated that tumor eradication *via* immunotherapy requires peripheral immune cell activity (15), and another study showed that peripheral immune activation is predictive of recovery times following surgery in humans (16). Also notably, in various types of cancers the relative amounts of platelets and neutrophils to lymphocytes appear to be superior predictive measures as compared to assessing each component independently (17–24). Some scoring systems such as the Glasgow Prognostic Score (GPS), which measures systemic inflammation by monitoring CRP and albumin, are effective at predicting overall survival (OS) in many solid organ malignancies (25). This review will discuss studies that utilized neutrophil-to-lymphocyte ratio (NLR), platelet-to-lymphocyte ratio (PLR), and CRP to determine their value for cancer detection and prognosis.

## Platelet-to-Lymphocyte Ratio

The PLR is defined as the relative number of platelets to lymphocytes, which, respectively, are hypothesized to have cancer-promoting and -fighting roles in circulation. Platelets are an integral part of hemostasis but have also been implicated to have a role in cancer progression. They can support cancer cell extravasation *via* the release of metalloproteases and promote tumor angiogenesis and growth at the metastatic site thought the release of growth factors, such as angiogenic factors, platelet-derived growth factor, and vascular endothelial growth factor (VEGF) (26), which enable tumor growth and metastatic spread (27–30). Platelets can also protect circulating tumor cells from killer T-cell-mediated cytolysis (31). In a symbiotic manner, cancer cells promote a platelet count increase and activation through the release of thrombopoietic cytokines and platelet agonists, respectively (9, 32, 33).

Lymphocytes are comprised of bone marrow-derived T and B cells. The B cell lineage produces antibodies that aid in the attack on invading bacteria, viruses, and toxins. T cells destroy host cells that been taken over by a virus or in select cases that have transformed to become cancerous. Lymphocytes have a crucial role in tumor defense by inducing cytotoxic death and inhibiting tumor cell proliferation and migration (5, 9).

The PLR has been shown to have predictive value in assessing the presence and progression of cancer and the response to drug therapy (34–36). In a meta-analysis of 18 studies which included 2,453 patients with ovarian cancer, PLR values were indicative of the stage of the disease and response to chemotherapy. The accuracy of predictions in these studies ranged from 55 to 80% (34). The variation in the performance was mainly attributed to the chosen cutoff for PLR. Although Polat et al. found an optimal cutoff for PLR to be 144.3 with 54% sensitivity and 59% specificity for malignancy prediction (37), Bakacak et al. found an optimal cut off to be 161.13 with 81.8% sensitivity and 50.8% specificity (38).

Platelet-to-lymphocyte has been shown to be a useful metric for determining the prognosis of a variety of cancers in patients. Increased PLR levels are a significant prognostic factor for poor prognosis in terms of OS in patients with gastric, colorectal, ovarian, hepatocellular, and lung cancers, some of which are summarized in Table 1 (18, 39–44). Raungkaewamee et al. observed that patients with a PLR of less than 200 had improved survival [progression-free survival (PFS), *p* = 0.003 and OS, *p* = 0.002] (42). However, the prognostic value of PLR remains controversial, as it failed to predict OS in several other studies (45–47). Xu et al. conducted a meta-analysis on PLR in patients with gastric cancer. Though the PLR was correlated with a higher risk of lymph node metastasis [odds ratio (OR) 1.5, 95% CI: 1.24–1.82] and increased the advanced stage cancer risk with (OR 1.99, 95% CI: 1.60–2.46), it was not a reliable predictor of OS, with an HR of 0.99 (0% CI: 0.9–1.1) (47).

**Table 1:** Clinical studies investigating the role of PLR in predicting cancer patient outcomes.

Reference	Cancer type	# of patients	Range cutoffs (survival)	Multivariate HR (OS)	Time of measurement
Wang et al. (39)	Prostate	290	PLR > 117.58	1.65, *p* = 0.044	Diagnosis
Yu et al. (40)	Non-small cell lung	210	PLR > 152.6	2.03, *p* < 0.001	Pretreatment
Krenn-Pilko et al. (41)	Breast	793	PLR > 292	1.92, *p* = 0.047	Preoperative
Raungkaewmanee et al. (42)	Epithelial ovarian	166	PLR > 200	NS	Preoperative
Ozawa et al. (43)	Colorectal	234	PLR > 25.4	CSS—3.61, *p* = 0.038	Preoperative
Szkandera et al. (20)	Colon	372	PLR > 224	NS	Preoperative
Smith et al. (44)	Pancreatic ductal adenocarcinoma	110	PLR > 150	1.00, *p* = 0.003	Preoperative

*CSS, cancer-specific survival; NS, not significant; OS, overall survival*.

## Neutrophil-to-Lymphocyte Ratio

The NLR is a measurement of the relative number of neutrophils to lymphocytes in the peripheral blood and is simply derived from blood tests by dividing the absolute number of circulating neutrophils by the circulating lymphocyte population per volume. A few studies have also reported a derived NLR (d-NLR), which is the neutrophil count divided by the result of white cell count minus neutrophil count (48–51). Neutrophils play an essential role in frontline defense against pathogens by initiating and amplifying inflammatory reactions. Analogously to platelets, neutrophils can also interact with cancer cells, and produce cytokines and effector molecules such as VEGF that stimulate tumor angiogenesis, growth, and metastasis (52–56). Activated neutrophils can migrate from the venous system to tumor niches where they release large amounts of reactive oxygen species that can induce cell DNA damage and genetic instability giving rise to adenomas in the cancer microenvironment (5, 57). Interestingly, neutrophils can also have antitumor roles through direct and antibody-mediated cytotoxicity of tumor cells and activation of immune cell types such as T-cells and dendritic cells. Neutrophils can be activated by cancer cell-derived cytokines including myeloid growth factors, tumor necrosis factor (TNF) alpha, IL-10, IL-6, granulocyte colony-stimulating factors, and transforming growth factor beta (58–63). In murine models, inactivation of the tumor suppressor gene STK11/LKB1 led to significant increases in tumor-promoting cytokines and neutrophil activity; treatment of mice with an IL-6 antibody decreased the degree of tumor-associated neutrophils and improved survival as compared to control mice (64). Another insidious contribution of neutrophils is that they can encourage cancer-associated thrombosis through the generation of neutrophil extracellular traps (NETs), which are web-like structures composed of DNA, histones, and granule proteins (65). NETs have been shown to contribute to venous thrombosis in animal models (66–68). Yet these same NETs have been found to have an anti-cancer effect, as they can also serve as primers for T-cells (69).

An elevated NLR is associated with advanced stage cancer, high histological grade, and metastasis (70); moreover, an elevated NLR is also an independent prognostic factor of coronary artery disease, hypertension, chronic kidney disease, diabetes, heart failure, cerebrovascular disease, and peripheral arterial disease (71). NLR has been shown to be sensitive to atherosclerotic risk factors such as smoking, alcohol consumption, hypercholesterolemia, and metabolic syndrome (71).

According to some published works, NLR is a discriminating factor for distinguishing between malignant borderline and benign tumors (34, 37, 72, 73), particularly in ovarian cancer patients. A systematic review explored 18 studies involving 3,453 patients and found NLR values in ovarian cancer patients are significantly different from healthy controls, but the diagnostic accuracy remains limited. For example, in 63 patients who underwent laparotomy for adnexal mass evaluation, it was found that the NLR was significantly higher in malignant cases compared to benign cases (*p* < 0.05) and that the NLR had high sensitivity in detecting mucinous cancers (78%) (72). Another study of 275 women with ovarian masses (borderline, malignant, and benign) found that the NLR was a significant predictor (AUC = 0.604, *p* = 0.02) in malignant cases but not in borderline tumors (37). Cho et al. studied 192 patients with epithelial ovarian cancer, 173 with benign ovarian tumors, and 405 healthy controls and found that preoperative NLR was significantly higher (mean, 6.02) than that in benign ovarian tumor subjects (mean 2.57) and healthy controls (mean 1.98). The sensitivity and specificity of NLR in discriminating ovarian cancer was 66.1% (95% CI: 59.52–72.68%) and 82.7% (95% CI: 79.02–86.38%) (cutoff value: 2.60) (74).

Neutrophil-to-lymphocyte is also an established indicator of PFS and OS in multiple types of cancers. Clinical studies investigating NLR as a predictor of cancer patient survival are summarized in Table 2 (17, 50, 75–79) In a notable study of 12,118 patients with multiple types of cancers, reduced OS was associated with an NLR > 4 (HR—1.57, *p* < 0.001) or dNLR > 2 (HR—1.54, *p* < 0.001) prior to the diagnosis (50). NLR has also been useful in informing patient mortality following treatment. Nakamura et al. demonstrated that an NLR > 3.91 prior to chemotherapy was associated with increased mortality in the next 100 days (80). Yet there are many studies that have found no association between NLR and survival (42, 81). Disagreement in the literature could be explained by the biphasic roles of neutrophils and other inconsistencies that will be surveyed in the forthcoming “Potential Utility of Blood Measurements” section.

**Table 2:** Clinical studies investigating the role of NLR in predicting cancer patient outcomes.

Reference	Cancer type	Measurements	# of patients	Range cutoffs	Multivariate HR (OS)	Time of measurement
Absenger et al. (17)	Colon	NLR	504	NLR > 4	1.95, *p* = 0.006	Preoperative
Azab et al. (75)	Breast	NLR	316	NLR > 3.3	4.07, *p* = 0.002	Pretreatment
Jung et al. (76)	Gastric	NLR	293	NLR > 2.0	1.61, *p* = 0.006	Presurgery
Pichler et al. (128)	Renal cell carcinoma	NLR	678	NLR > 3.3	1.59, *p* = 0.014 CSS—1.39, *p* = 0.184	Pretreatment
Proctor et al. (50)	Multiple	NLR dNLR	12,118	NLR > 4dNLR > 2	Prediagnosis—NLR—1.57, *p* < 0.001, dNLR—1.54, *p* < 0.001 Postdiagnosis—NLR—1.86, *p* < 0.001, dNLR—1.76, *p* < 0.001	Within 2 years of diagnosis
Rachidi et al. (77)	Head and neck	NLR	543	NLR in highest tertile	2.39, *p* = 0.0001	Pretreatment
Ubukata et al. (78)	Gastric	NLR	157	NLR > 5	RR—5.78, *p* < 0.001 (presurgery) RR—3.26, *p* = 0.540 (postsurgery)	Presurgery and 14 days post
Viers et al. (79)	Clear cell carcinoma	NLR	827	NLR > 4	1.02, *p* < 0.01	Pretreatment

*CSS, cancer-specific survival; OS, overall survival; RR, relative risk*.

## C-Reactive Protein

C-reactive protein is an acute phase non-specific inflammatory reactant that can be elevated due to an infection, invasive procedure, or medications (82, 83). CRP is released by the liver in response to increased levels of IL-6 released by activated macrophages, though it has also been found that IL-1 and TNF can also stimulate CRP synthesis (83). Additionally, tumor cells have been proposed as a potential trigger for CRP upregulation, as they secrete IL-6 and 8 to stimulate liver CRP production (5). CRP has a half-life of 19 h in the circulation and is a relatively stable marker of inflammation in comparison to select cytokines, which only have half-lives on the order of minutes (83). The GPS assigns a score of 0 to patients with a CRP < 10 mg/L and albumin ≥ 35 g/L, score of 1 to those with CRP ≥ 10 mg/L and albumin < 35 g/L and score of 2 for patients with CRP > 10 mg/L and albumin < 35 g/L. The modified Glasgow Prognostic Score (mGPS) is the same except a score of 1 is only dependent on having a CRP is greater than 10 mg/L (84–86).

GPS and mGPS descriptors have been shown to have a prognostic significance in patients with solid tumors, especially in gastrointestinal and kidney-related malignancies (87–92). Many of these clinical studies investigating the prognostic value of CRP are listed in Table 3 (93–100). It was also found that in pancreatic cancer patients, there is an association of only elevated CRP levels with poor clinical outcomes (OR: 1.6, 95% CI: 1.16–2.21) in a multivariate analysis (93). Furthermore, there is strong positive correlation between CRP and IL-6 levels found in advanced pancreatic cancer patients (101). Yet, there is not total agreement in the literature over the prognostic utility of CRP in select cancer types. In a meta-analysis, only three of six studies found low CRP levels to be associated with increased survival in pancreatic cancer. There is wide variation in cutoffs considered for these prognosis studies—around 34% used a CRP cutoff of <10 mg/L while other studies spanned from 2 to 50 mg/L CRP. The reason there may exist disparate cutoffs may be in part due to the highly variable nature of the peripheral blood-derived CRP levels. In order to reduce the amount of variability of CRP measurements, some researchers have instead quantified tumoral CRP over serum CRP for prognosis and recurrence predictions (102). There remains a need for larger studies to be conducted to define the importance of CRP as a biomarker for informing cancer staging and prognosis.

**Table 3:** Clinical studies investigating the role of CRP in predicting cancer patient outcomes.

Reference	Cancer type	Measurements	# of patients	Range cutoffs	Multivariate HR (OS)	Time of measurement
Szkandera et al. (93)	Pancreatic	CRP	474	CRP > 4.5 mg/L	1.6, *p* = 0.005	Diagnosis
Lamb et al. (94)	Clear cell RCC	mGPS	169	mGPS^a^	4.59, *p* < 0.001	Pretreatment
Komai et al. (95)	RCC	CRP	101	CRP > 5 mg/L	CSS—2.7, *p* = 0.012	Presurgery
Karakiewicz et al. (96)	RCC	CRP	313	CRP > 23 mg/L	CSS—11, *p* = 0.002	Preoperative
Crumley et al. (97)	Gastric	CRP, alb	217	CRP > 10 mg/L, alb < 35 g/L	Alb—NS, CRP—2.37, *p* < 0.001	Pretreatment
Tatokoro et al. (98)	RCC	CRP	40	CRP > 5 mg/L	CSS—4.84 (non-normalized postsurgery), *p* < 0.0001	Pre/postoperative
Shimada et al. (99)	Esophageal squamous cell carcinoma	CRP	150	CRP > 1 mg/L	1.68, *p* = 0.049	Preoperative
Shiu et al. (100)	Colorectal	CRP	212	CRP > 5 mg/L	CSS—6.51, *p* = 0.016	Preoperative

*^a^MGPS = 1 if CRP > 10 mg/mL, mGPS = 2 if CRP > 10 mg/L and albumin < 35 g/L, mGPS = 0 if both CRP and albumin are normal*.

*alb, albumin; CRP, C-reactive protein; CSS, cancer-specific survival; OS, overall survival; RCC, renal cell carcinoma*.

## Multifactorial Analyses of CBC Parameters

Several studies have considered multiple parameters of CBC counts; some of which are summarized in Table 4 (1, 103–114). A few multifactorial analyses found just a single inflammation-related parameter that was an independent predictor of prognosis, which for the most part was NLR (51, 104, 106, 113, 115). For example, He et al. analyzed PLR and NLR in 243 patients with metastatic colorectal cancer and found that only NLR was a significant predictor for OS and disease-free survival (DFS). Similarly, Jia et al. investigated the prognostic significance of peripheral blood NLR, PLR, LMR, and individual platelet counts, lymphocytes, neutrophils, and monocytes individually for predicting DFS and OS in patients with breast cancer of different molecular subtypes. Multivariate analysis revealed that only NLR was a significant determinant of DFS and OS in all breast cancer patients (DFS, HR = 1.50 95% CI: 1.14–1.97, *p* = 0.004; OS, HR = 1.63, 95% CI: 1.07–2.49, *p* = 0.022) and in triple negative (negative for estrogen receptor, progesterone receptor, and human epidermal growth factor 2 receptor) breast cancer patients (DFS, HR = 2.58, 95% CI: 1.23–5.42, *p* = 0.012; OS, HR = 3.05, 95% CI: 1.08–8.61, *p* = 0.035) (106). Another study compared NLR, d-NLR, PLR, and LMR for predicting clinical outcome in 205 surgical colorectal cancer patients. Only elevated NLR was significantly associated with reduced PFS and OS in (RFS, HR 2.52, 95% CI: 1.65–3.83, *p* < 0.001; OS, HR 2.73, 95% CI: 1.74–4.29, *p* < 0.001) (51). A few studies found other CBC components that were useful outside of NLR. Some studies reported that mGPS but not NLR or PLR were independent prognostic factors of survival outcomes in surgically treated cancer patients (116, 117). Overall, even with the incorporation of CBC counts into multivariate analyses, mostly NLR remains significant. It remains to be determined whether the remaining insignificant covariates provide overlapping information with the significant covariate, or offer no prognostic value.

**Table 4:** Clinical studies investigating the role of multiple blood measurements in predicting cancer patient outcomes.

Reference	Cancer type	Measurements	# of patients	Range cutoffs (survival)	Multivariate HR (OS)	Time of measurement
Aizawa et al. (103)	Gastric	NLR, CRP, Hb, Plt, Alb, lym, alb	262	NLR > 3.2, Hb < 13 g/dL, Plt > 250 K/µL, CRP > 1 mg/dL, alb < 35 g/L	ORs; Hb-1.89, *p* = 0.48; NLR—2.21, *p* = 0.012	Baseline preoperative
Deng et al. (104)	Gastric	NLR, dNLR, PLR, LMR	385	NLR > 2.36, dNLR > 1.85, PLR > 132, LMR > 4.95	All NS except dNLR—1.42, *p* = 0.012	Preoperative
Guthrie et al. (1)	Colorectal	mGPS, NLR	206	mGPS,^a^ NLR > 5	mGPS—1.97, *p* < 0.05 NLR—3.07, *p* < 0.05	Preoperative
Hsueh et al. (105)	Laryngeal squamous cell cancer	NLR, PLR, LMR, plt, neut, lym, monocytes		NLR > 2.4, PLR > 111, LMR < 3.5, lym < 1.6 K/µL, neutr > 4.3 K/µL, plt > 200 K/µL	CSS: Lymph—2.20, *p* < 0.0001; NLR—1.84, *p* = 0.001, PLR—1.71, *p* = 0.002, LMR—1.98, *p* < 0.001	Preoperative
Jia et al. (106)	Breast	NLR, LMR	1,570	NLR < 2, LMR > 4.8	NLR—1.63, *p* = 0.022	Pretreatment
Kang et al. (107)	Non-small cell lung	NLR, PLR	187	NLR > 4, PLR > 160	NLR—1.47, *p* = 0.043; PLR—NS	Pretreatment
Kim et al. (45)	Gastric	NLR, PLR	1,986	NLR > 2, PLR > 126	NLR—1.4, *p* = 0.023 PLR—NS	Presurgery
Kim et al. (108)	Ovarian clear cell carcinoma	NLR, PLR, LMR	109	NLR < 2.4, PLR < 178.3, MLR > 0.2, neut > 4.37 K/µL, plt > 300 K/µL, mono > 0.39 K/µL	All are NS in OS	Pretreatment
Kinoshita et al. (109)	Hepatocellular carcinoma	GPS, mGPS, NLR, PLR	150	GPS,^b^ mGPS,^a^ NLR > 5, PLR^c^	All NS except GPS—1.78, *p* = 0.002	Pretreatment
Leitch et al. (110)	Colorectal	NLR, monocyte, mGPS	149	NLR > 5, mono > 0.9 K/µL, mGPS^a^	NLR-NS, monocyte—3.79, *p* = 0.015, mGPS—2.21, *p* = 0.024	Presurgery
Martin et al. (111)	Advanced pancreatic	NLR, PLR, mGPS	124	NLR > 5, PLR > 200mGPS^a^	NLR—1.6, *p* = 0.02; PLR—1.58, *p* = 0.02; mGPS—1.41 *p* = 0.01	During treatment
Oh et al. (112)	Heptocellular carcinoma	CRP, NLR	318	CRP > 6.3 mg/L, NLR > 2.3	CRP—1.52, *p* = 0.027, NLR—1.60, *p* = 0.009	Pretreatment
Stotz et al. (113)	Pancreatic	PLR, NLR, mGPS	371	PLR > 150, NLR > 5, mGPS^a^	PLR—NS, NLR—2.53, *p* < 0.001 (inoperable), NLR—1.61, *p* = 0.039 (operable), mGPS—NS	Pretreatment
Wang et al. (39)	Gastric	GPS, PLR, NLR, neut, plt, CRP, alb	324	GPS,^b^ PLR,^c^ NLR > 5, neut > 7.5 K/µL, plt > 400 K/µL, CRP > 10 mg/L, alb < 35 g/L	All-NS except GPS—1.40, *p* = 0.014	Preoperative

*^a^MGPS = 1 if CRP > 10 mg/L, mGPS = 2 if CRP > 10 mg/L and alb < 35 g/L, mGPS = 0 if both CRP and alb are normal*.

*^b^GPS = 1 if CRP > 10 mg/L or alb < 35 g/L, GPS = 2 if CRP > 10 mg/L and alb < 35 g/L, GPS = 0 if both CRP and alb are normal*.

*^c^PLR = 1 if PLR > 150, PLR = 2 if PLR > 300, PLR = 0 if PLR < 150*.

*alb, albumin; CRP, C-reactive protein; plt, platelet; neut, neutrophil; lym, lymphocyte; mono, monocyte; OR, odds ratio; CSS, cancer-specific survival; Hb, hemoglobin; OS, overall survival; LMR, lymphocyte-to-monocyte ratio*.

Other studies have found that multiple components of CBCs offer unique information that contribute to improved prediction of patient prognosis (105, 118, 119). Hsueh et al. investigated the prognostic value of preoperative counts of neutrophils, platelets, lymphocytes, and monocytes and ratios in 979 patients with laryngeal squamous cell cancer and measured prediction success of DFS and cancer-specific survival (CSS). They found that patients in the highest tertile of NLR (>2.4) and PLR (>111) were at higher risk of reduced DFS and CSS when compared using a multivariate analysis. The lowest tertile of lymphocytes (<1.6 × 10^9^/L) and LMR (<3.5) were also associated with high risk of poor outcomes. Stotz et al. utilized several biomarkers such as hemoglobin, white blood cell count, NLR, d-NLR, LMR, and PLR in their prediction model for disease recurrence and survival in 149 gastrointestinal stroma tumor patients after surgical resection (19) and found significance in low hemoglobin (*p* = 0.029) and elevations in WBCs (*p* = 0.004), NLR (*p* = 0.015), and d-NLR (*p* = 0.037) being associated with poor OS in patients. Low Hb (0.049), WBC (*p* = 0.001), and elevated NLR (*p* = 0.007), d-NLR (*p* = 0.043), and PLR (*p* = 0.024) was associated with decreased RFS.

The majority of the studies mentioned up to this point have relied on single time point measurements around the time of treatment or diagnosis. In the next section, we discuss studies that have tracked inflammatory laboratory counts over multiple time points in patients and used this information to improve patient prognosis predictions.

## Longitudinal Studies

Longitudinal trends of laboratory counts have primarily been accounted for by using counts prior to and after the diagnosis or treatment of a disease for prognosis prediction for patients with cancer and other malignancies. Guthrie et al. utilized longitudinal measurements of mGPS and NLR in colorectal cancer patients undergoing a curative resection. Measurements were accounted for at multiple time points prior to and following the operation. They discovered that preoperative mGPS and NLR and only the postoperative mGPS were independent predictors of prognosis (1). Derman et al. also monitored NLR in advanced non-small lung cell cancer patients before and 6 and 12 weeks after chemotherapy. The median NLR was 3.6 (range 0.1898–30.910) at baseline, 3.11 (range 0.2703–42.11) at 6 weeks and at 12 weeks it was 3.52 (range 0.2147–42.93) (120). A higher NLR at baseline, 6 and 12 weeks was associated with decreased OS.

In additional to monitoring laboratory counts surrounding a treatment, longitudinal measures could also be tracked in a trajectory analysis over various length scales and at other stages of the disease to determine if dynamic biological changes are indicative of a patient’s prognosis. One study demonstrated that the relative temporal changes of various blood cell parameters over a few days showed up in patterns that could uniquely identify different responses to infections in a mouse model (121). Additionally, it has been demonstrated that trajectories of body mass index over a lifetime are informative in improving prognosis predictions of prostate cancer patients (122). The same techniques could be applied with inflammation-related data and cancer, ideally prior to cancer treatment or diagnosis, before interventions significantly affect the lab counts.

## Potential Utility of Blood Measurements

It is evident that these inflammation-related blood measurements can provide prognostic information. However, even though there have been several studies published on the prognostic significance of these factors in multiple types of cancers, they are not routinely used in a clinical setting and it remains unclear how to incorporate these data into management of patients with cancer. This could possibly be attributed to discrepancies and limitations found among previous studies. Meta-analyses of numerous studies have found inconsistent results relating to the prognostic value of blood cell parameters as predictors of survival. For example, Stevens et al. found that NLR and CRP only showed significance in two of eight articles and three of six articles, respectively (81). These inconsistencies might be due to the fact that the majority of the studies are retrospective, contain a small number of patients, and reflect heterogeneous populations. Blood cell population heterogeneity increases with patient age, nature of treatments performed, and comorbidities such as active infection, cardiovascular disease, pulmonary disease, and obesity, which are also associated with increased inflammatory markers (123–125). Additionally, the cancer cell histologic classification could greatly influence the variation reported within these studies. A meta-analysis on renal cell carcinoma (RCC) patients found heterogeneity in the types of populations recorded; two articles looked at clear-cell RCC and another article focused on non-clear cell RCC (126). It was also difficult to find consensus among studies that investigated mGPS and NLR due to heterogeneity of stages within the populations, four studies had patients with T1-T4 disease (79, 94, 127, 128), two studies with T1-T3 (129, 130), and two studies did not include T stage (131, 132). Moreover, the cutoff values that were applied for PLR-, NLR-, and CRP-related measurements were not standardized (21, 79, 126, 128). Cutoff values were instead chosen *via* a sensitivity analysis to maximize the receiver operating characteristic area under the curve. This method only ensures high prediction performance in a specific cohort from which the study was done and may not be generalizable to a larger population, making it less feasible for study validation or translation of these results to a clinical setting. Eventually, many of these issues could be resolved by planning and executing more carefully planned prospective studies with standardized cutoff values.

Observational studies on the blood measurements in cancer patients are subject to limitations. One concern would be that there is selection bias in the population that is mostly likely to have their laboratory counts measured. This could be especially problematic in longitudinal studies, in which cohorts are further filtered to patients that have measurements within a time period of interest. It is critical to consider that retrospective data has not been collected with the intent of creating a dataset for research. The CBC is typically ordered to diagnose a condition, screen for a condition, or monitor for preexisting conditions. Rimma et al. found that the laboratory’s test numerical value and rate of testing were correlated features, likely because when a value is outside of the normal range, additional testing is prompted until the value returns to a normal range (133). Additionally, another inherent problem is that the bias of missing data are not random; patients are seen and measured more often when they are sick, and measured less when they are healthy. Dynamics of certain lab tests vary; some are only measured in mixed patterns and biases can be disambiguated by accounting for the laboratory measurement frequency (133). One way to overcome measurements related to interventions would be algorithmically filtering laboratory values that have been taken in quick succession. Another limitation is that the models being used in these studies are not adequately equipped to deal with multiple components and time series analysis, in which there could be a substantial amount of information on the pathophysiological progression of the disease. Many of these limitations could be overcome by prospective studies as mentioned before, and employing computational methods that can handle missing data, remove noise and autocorrelation, handle larger population sizes, and extract complicated relationships among the data.

## Leveraging Laboratory Data with Computational Models

Computational and mathematical models have been developed to address the complexity of high-dimensional biological data and potentially give some insight into the biological mechanisms of the disease. Two common types of computational modeling approaches include: (1) statistical and artificial intelligence (AI)based models, which reproduce a response with a given set of variables and all variables and/or parameters carry some uncertainty and (2) deterministic model which represent chemical and physical phenomenon in which there are some known relationships among the states and events (134).

A subset of methods within the field of machine learning/AI use deep learning, which is inspired by information processing in biological nervous systems. These tools can perform classification tasks similarly to an expert of a particular field and have been successful in subjects such as computer vision, speech recognition, bioinformatics, and natural language processing. These techniques are also being applied with longitudinal electronic health record (EHR) data. For example, Choi et al. used a longitudinal sequence of serum uric acid that were episodic in nature and accurately distinguished signatures of gout versus acute leukemia. Lipton et al. demonstrated that Recurrent Neural Networks, particularly those that use Long Short-Term Memory hidden units, are effective models for learning sequence data (135). These types of neural networks effectively model varying length sequences and capture long range dependencies. Even though EHR data are plagued with noisy and sparse data with irregularly timed observations, there are a variety of interpolation techniques employed in deep learning techniques to fill in missing data such as Gaussian process regression (136).

Mathematical modeling of the governing biochemical and biophysical phenomenon may also be instrumental in making cancer patient predictions. In one study, it was shown that an *in silico* framework based on cellular signaling interactions could predict the influence of eight different cytokines on the survival, duplication and differentiation of CD133^+^ hematopoietic stem and progenitor cells, which are essential for hematopoietic stem cell transplantation (137). This simulation which accounted for individual and combined effects of the cytokines was developed and validated with literature searches and *in vitro* experimental models. Computational models have also been instrumental in accounting for the combinatorial complexity required for melanoma drug discovery (138). These methods could be explored further when determining the best possible combination of biomarkers/cell counts to explore in a multivariate analysis.

## Conclusion

In this review, we described the utility of inflammatory markers to improve cancer staging and prognosis predictions. We discussed the relationship between inflammation and cancer and described the notable blood measurements, such as platelets, neutrophils, lymphocytes, their relative ratios, and CRP. These studies are subject to limitations and inconsistencies, but with the addition of higher resolution data (multivariate and temporal), prospective studies, and advanced computational methods will have the potential to improve model predictions and yield a more thorough understanding of the pathophysiology of the disease.

## Author Contributions

JS provided the initial conception, design, and writing of the article. AM and MA provided feedback, writing, and references for sections that involved mechanistic biology of platelets and immune cells, respectively. GT, JS, ST, and JL are all clinicians and each provided clinical research references, additional writing, and knowledge on current clinical care of cancer patients. PM and TW provided references, editing, and knowledge on statistics and new machine learning techniques to leverage this type of data. OM helped edit and write general sections of the article.

## Conflict of Interest Statement

The authors declare that the research was conducted in the absence of any commercial or financial relationships that could be construed as a potential conflict of interest.
